# Syntheses and crystal structures of two piperine derivatives

**DOI:** 10.1107/S2056989020004648

**Published:** 2020-04-09

**Authors:** Toshinari Ezawa, Yutaka Inoue, Isamu Murata, Mitsuaki Suzuki, Koichi Takao, Yoshiaki Sugita, Ikuo Kanamoto

**Affiliations:** a Faculty of Pharmacy and Pharmaceutical Science, Josai University, 1-1 Keyakidai, Sakado-shi, Saitama, 3500295, Japan; b Faculty of Science, Josai University, 1-1 Keyakidai, Sakado-shi, Saitama, 3500295, Japan

**Keywords:** crystal structure, organic crystal, piperine, hydrogen bond

## Abstract

The title compounds, 5-(2*H*-1,3-benzodioxol-5-yl)-*N*-cyclo­hexyl­penta-2,4-dienamide, C_18_H_21_NO_3_ (I), and 5-(2*H*-1,3-benzodioxol-5-yl)-1-(pyrrolidin-1-yl)penta-2,4-dien-1-one C_16_H_17_NO_3_ (II), are derivatives of piperine, which is known as a pungent component of pepper. Their geometrical parameters are similar to those of the three polymorphs of piperine, which indicate conjugation of electrons over the length of the mol­ecules. The extended structure of (I) features N—H⋯O amide hydrogen bonds, which generate *C*(4) [010] chains. The crystal of (II) features aromatic π–π stacking, as for two of three known piperine polymorphs.

## Abstract 2

The title compounds, 5-(2*H*-1,3-benzodioxol-5-yl)-*N*-cyclo­hexyl­penta-2,4-dienamide, (I), and 5-(2*H*-1,3-benzodioxol-5-yl)-1-(pyrrolidin-1-yl)penta-2,4-dien-1-one (II), are derivatives of piperine, which is known as a pungent component of pepper. Their geometrical parameters are similar to those of the three polymorphs of piperine, which indicate conjugation of electrons over the length of the mol­ecules. The extended structure of (I) features N—H⋯O amide hydrogen bonds, which generate *C*(4) [010] chains. The crystal of (II) features aromatic π–π stacking, as for two of three known piperine polymorphs.

## Chemical context   

**Figure 1:**
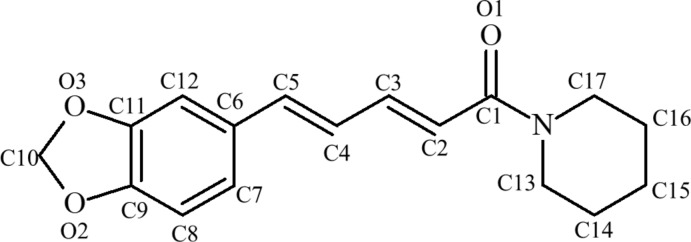
The chemical structure of piperine.

Piperine [(2*E*,4*E*)-1-[5-(1,3-benzodioxol-5­yl)-1-oxo-2,4-pen­ta­dien­yl]piperidine, C_17_H_19_NO_3_, is the major pungent ingredient of Piperaceae pepper (*Piper nigrum*). Piperine is an amide having a methyl­ene­dioxy­phenyl grouping as a characteristic of its chemical structure (Fig. 1 ▸). Inter­estingly, when the amide group is in a near planar conformation, the conjugated state of the penta­diene chain of piperine has the property that electrons are easily donated and the stretching vibration of the amide carbonyl group is affected (Pfund *et al.*, 2015 ▸). As part of our studies in this area, we have already reported a complex using the poorly water-soluble piperine (log *P* = 2.25) and the cyclic polysaccharide cyclo­dextrin (Szejtli, 1998 ▸; Ezawa *et al.*, 2016 ▸). In addition, piperine has been evaluated for its inclusion mechanism and dissolution properties using various cyclo­dextrins (Ezawa *et al.*, 2018 ▸, 2019 ▸). The synthesis of piperine derivatives was necessary to understand the inclusion mechanism of piperine and cyclo­dextrin and the detailed mol­ecular behaviour of piperine.
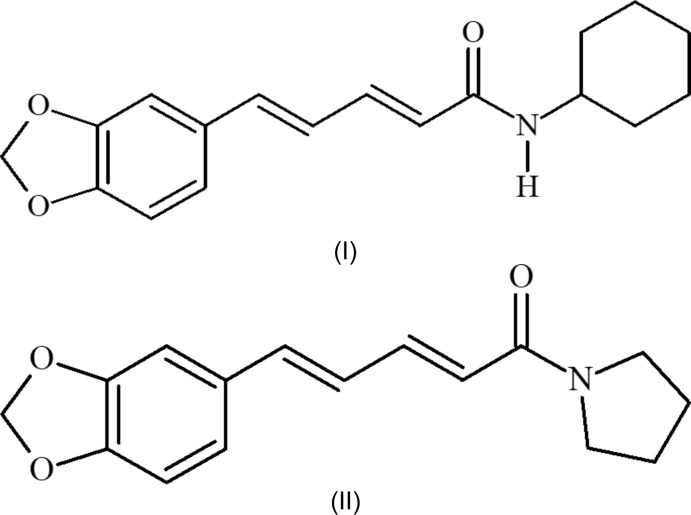


Therefore, the aim of this study was to synthesize the title compounds (2*E*,4*E*)-5-(2*H*-1,3-benzodioxol-5-yl)-*N*-cyclo­hexyl­penta-2,4-dienamide, C_18_H_21_NO_3_, (I), and (2*E*,4*E*)-5-(2*H*-1,3-benzodioxol-5-yl)-1-(pyrrolidin-1-yl)penta-2,4-dien-1-one, C_16_H_17_NO_3_, (II), from piperine and to determine their X-ray crystal structures. The log *P* of (I) is 3.36 and that of (II) is 2.36. Assessing the structural properties of the title compounds (crystal structure, geometry, inter­molecular inter­actions, *etc*.) will help to evaluate the inclusion behaviour of piperine with cyclo­dextrin.

## Structural commentary   

**Figure 2:**
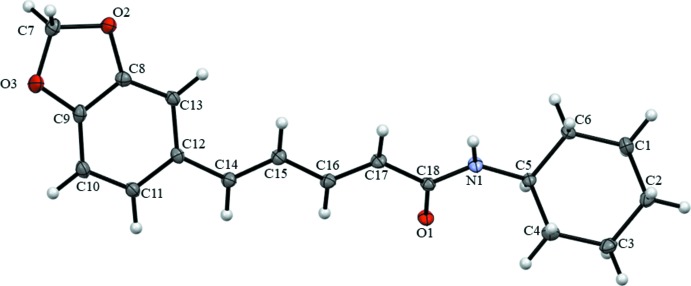
Displacement ellipsoid drawing at a 50% probability level of the asymmetric unit of (I).

Compound (I) (Fig. 2 ▸) crystallizes in the monoclinic space group *P*2_1_/*c* with four mol­ecules per unit cell. The C1–C6 cyclo­hexyl ring adopts a chair conformation with the exocyclic C5—N1 bond in an equatorial orientation. The C7–C12/O2/O3 fused-ring system is almost planar (r.m.s. deviation = 0.020 Å) and subtends a dihedral angle of 21.57 (4)° with the cyclo­hexyl ring. The bond distances and angles (amide, penta­diene and methyl­ene­dioxy­phenyl moieties) of (I) are not significantly different from the equivalent data for the three polymorphs of piperine (Pfund *et al.*, 2015 ▸) (Table 1 ▸).

**Figure 3:**
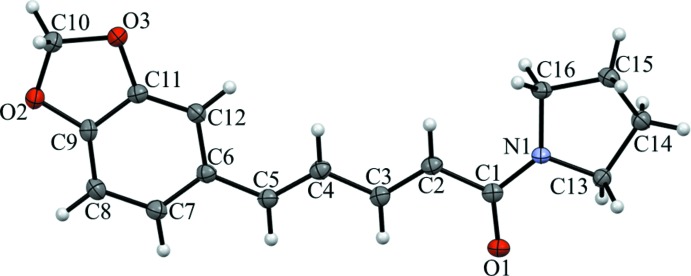
Displacement ellipsoid drawing at a 50% probability level of the asymmetric unit of (II).

Compound (II) (Fig. 3 ▸), also known as piperilyn, crystallizes in the ortho­rhom­bic space group *Pbca* with eight mol­ecules per unit cell. The C13–C16/N1 ring is well described as being twisted with C14 and C15 deviating from C13/N1/C16 by 0.205 (2) and −0.382 (2) Å, respectively. The C9/O2/C10/O3/C11 ring has a clear tendency towards an envelope conformation [deviation of C10 from the other four atoms = −0.216 (2) Å]. The dihedral angle between the C13–C16/N1 and C6–C12/O2/O3 rings (all atoms) is 12.29 (10)°. As with (I), the key bond-distance data for (II) are comparable to those of piperine (Table 1 ▸).

Thus, we may conclude that the title compounds show intra­molecular resonance from the amide group to the ether O atoms of the methyl­ene­dioxy­phenyl moiety, similar to piperine.

## Supra­molecular features   

Piperine crystallizes in three polymorphs: form I [CCDC (Groom *et al.*, 2016) refcode: PIPINE10] and form II (PIPINE12) in space group *P*2_1_/*n* and form III (PIPINE13) in space group *C*2/*c* (Table 1 ▸) (Pfund *et al.*, 2015 ▸). The packing for forms II and III features aromatic π–π stacking inter­actions, while that of form I does not.

**Figure 4:**
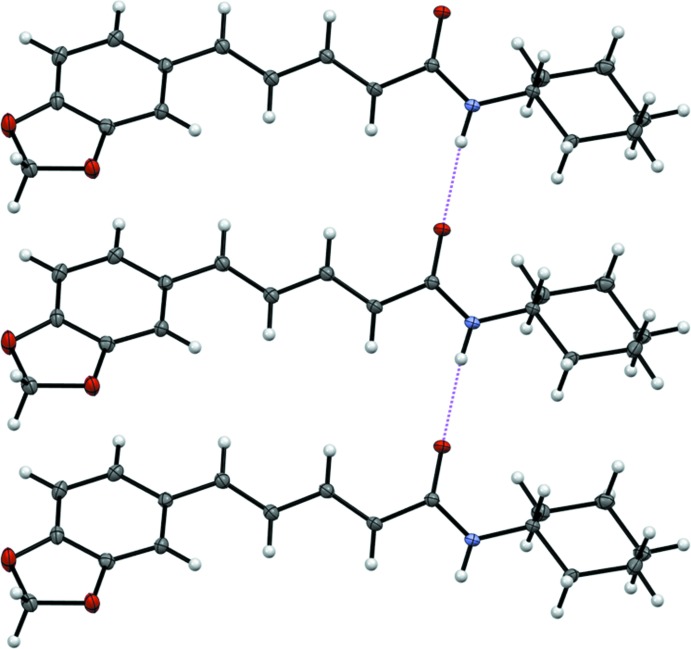
A view along the *c-*axis direction of the crystal packing of (I). The N—H⋯O hydrogen bonds are drawn as dashed lines.

**Figure 5:**
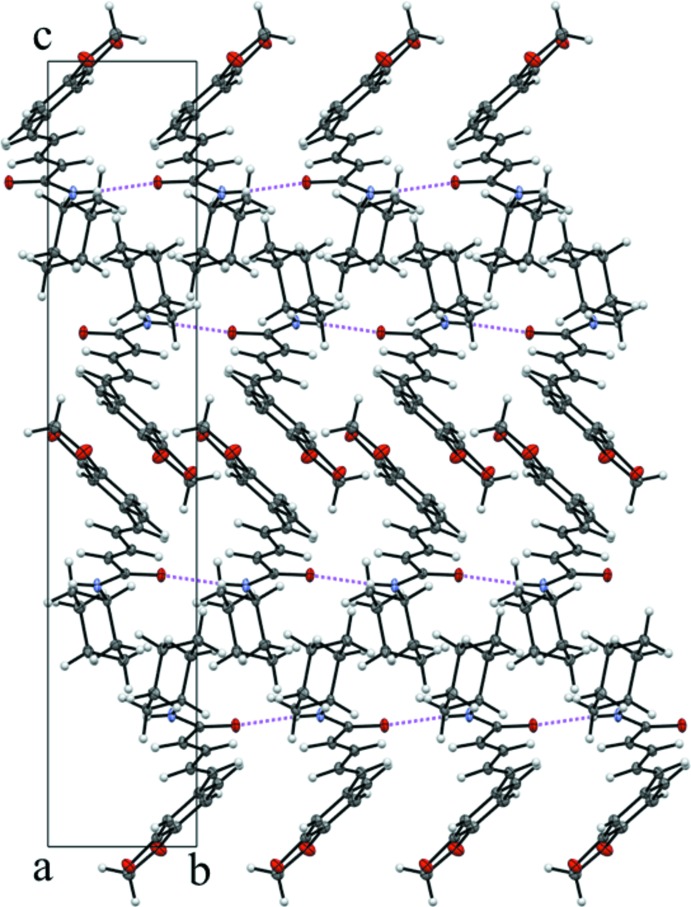
The unit-cell packing for (I) viewed down [100] with hydrogen bonds drawn as dashed lines.

The crystal structure of (I) does not feature π–π stacking inter­actions, which is similar to piperine form I. Compound (I) possesses an N—H grouping, which forms a classical N1—H⋯O1 hydrogen bond (Table 2 ▸) between the amide-bond sites, generating [010] *C*(4) chains (Fig. 4 ▸) with adjacent mol­ecules related by simple translation. The unit-cell packing for (I) is illustrated in Fig. 5 ▸.

**Figure 6:**
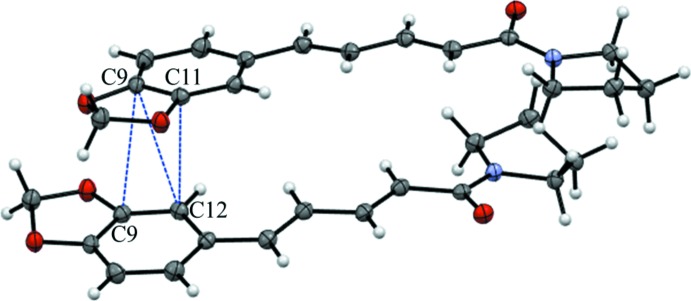
Fragment of the crystal of (II) showing close C⋯C contacts due to π–π stacking.

**Figure 7:**
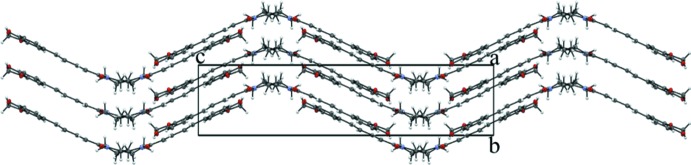
The unit-cell packing for (II) viewed down [100].

The structure of (II) does feature π–π stacking with the closest inter­molecular contacts being C9⋯C9 = 3.268 (3), C9⋯C12 = 3.322 (3) and C11⋯C12 = 3.287 (3) Å (Fig. 6 ▸). The overall packing for (II) can be described as undulating sheets propagating in the (010) plane (Fig. 7 ▸).

## Synthesis and crystallization   

Piperine was purchased from Fujifilm Wako Pure Chemical Co., Ltd. The synthesis of piperine derivatives was performed using a previously reported procedure (Takao *et al.*, 2015 ▸). After dissolving piperine in ethanol, hydrolysis was performed by stirring for 20 h in the presence of KOH. After evaporating the solvent under vacuum, the resulting reaction mixture was suspended in water and acidified with 4 *M* HCl to pH < 1. The resultant pale-brown precipitate was collected by filtration, washed with cold water and recrystallized from methanol solution to give piperic acid. The piperic acid (1.0 mmol) was dissolved in CH_2_Cl_2_ (5 ml) and oxalyl chloride (10 mmol) was added and the mixture was stirred at room temperature for 3 h. The solvent and excess oxalyl chloride were then evaporated under reduced pressure.

To prepare (I), the crude acid chloride generated was dissolved in CH_2_Cl_2_ (2 ml) and cyclo­hexyl­amine (1.2 mmol) and Et_3_N (8 mmol) were added, and the mixture was stirred at 273 K for 5 h. Ice-cold water was added to the mixture, followed by extraction with chloro­form (5 ml). The organic layer was dried over Na_2_SO_4_ and the solvent was evaporated under reduced pressure. The residue was purified by silica-gel column chromatography (eluent hexa­ne:etyl acetate 1:1 *v*/*v*) to give (I) in the form of a yellow powder. Light-yellow needles of (I) were recrystallized from ethyl acetate solution.

Compound (II) was prepared by the same procedure with pyrrolidine (1.2 mmol) replacing the cyclo­hexyl­amine to give (II) in the form of a white powder. Colourless needles of (II) were recrystallized from ethyl acetate solution.

## Refinement   

Crystal data, data collection and structure refinement details are summarized in Table 3 ▸. Hydrogen atoms for carbon atom were included in their calculated positions and refined as riding atoms with *U*_iso_(H) = 1.2*U*_eq_(C). The hydrogen atom attached to N1 in (I) was located in a difference-Fourier map and its position freely refined with *U*_iso_(H) = 1.2*U*_eq_(N).

## Supplementary Material

Crystal structure: contains datablock(s) I, II, global. DOI: 10.1107/S2056989020004648/hb7897sup1.cif

Structure factors: contains datablock(s) I. DOI: 10.1107/S2056989020004648/hb7897Isup2.hkl

Structure factors: contains datablock(s) II. DOI: 10.1107/S2056989020004648/hb7897IIsup3.hkl

Supporting information file. DOI: 10.1107/S2056989020004648/hb7897Isup4.cml

Supporting information file. DOI: 10.1107/S2056989020004648/hb7897IIsup5.cml

CCDC references: 1994582, 1994581

Additional supporting information:  crystallographic information; 3D view; checkCIF report

## Figures and Tables

**Table 1:** Key geometrical parameters (Å) for the title compounds and piperine polymorphs

	(I)	(II)	PIPINE10	PIPINE12	PIPINE13
Amide	C18—N1 (1.344)	C1—N1 (1.350)	C1—N1 (1.331)	C1—N1 (1.363)	C1—N1 (1.353)
	C18—O1 (1.242)	C1—O1 (1.243)	C1—O1 (1.218)	C1—O1 (1.235)	C1—O1 (1.482)
	C14—C15 (1.346)	C4—C5 (1.345)	C4—C5 (1.312)	C4—C5 (1.330)	C4—C5 (1.347)
Penta­diene	C15—C16 (1.444)	C3—C4 (1.441)	C3—C4 (1.437)	C3—C4 (1.440)	C3—C4 (1.442)
	C16—C17 (1.342)	C2—C3 (1.341)	C2—C3 (1.311)	C2—C3 (1.332)	C2—C3 (1.341)
	C17—C18 (1.479)	C1—C2 (1.480)	C1—C2 (1.473)	C1—C2 (1.477)	C1—C2 (1.482)
	C8—C9 (1.390)	C6—C7 (1.397)	C6—C7 (1.387)	C6—C7 (1.399)	C6—C7 (1.403)
	C8—C13 (1.371)	C6—C12 (1.412)	C6—C12 (1.396)	C6—C12 (1.414)	C6—C12 (1.412)
	C9—C10 (1.374)	C7—C8 (1.403)	C7—C8 (1.393)	C7—C8 (1.395)	C7—C8 (1.393)
Methyl­ene­dioxy­phen­yl	C10—C11 (1.402)	C8—C9 (1.369)	C8—C9 (1.343)	C8—C9 (1.360)	C8—C9 (1.371)
	C11—C12 (1.399)	C9—C11 (1.385)	C9—C11 (1.357)	C9—C11 (1.377)	C9—C11 (1.381)
	C12—C13 (1.412)	C11—C12 (1.364)	C11—C12 (1.364)	C11—C12 (1.370)	C11—C12 (1.367)
	C8—O2 (1.371)	C9—O2 (1.378)	C9—O2 (1.373)	c9—O2 (1.383)	C9—O2 (1.378)
	C9—O3 (1.370)	C11—O3 (1.376)	C11—O3 (1.362)	C11—O3 (1.383)	C11—O3 (1.383)
π-stacking close contacts		C9⋯C9 (3.268)		C8⋯C8 (3.110)	C9⋯C12 (3.327)
		C9⋯C12 (3.322)		C8⋯C8 (3.303)	
		C11⋯C12 (3.287)			

**Table 2:** Hydrogen-bond geometry (Å, °) for (I)

*D*—H⋯*A*	*D*—H	H⋯*A*	*D*⋯*A*	*D*—H⋯*A*
N1—H1⋯O1^i^	0.874 (16)	2.086 (16)	2.9547 (12)	172.8 (14)

Symmetry code: (i) 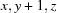.

**Table 3:** Experimental details

	(I)	(II)
Crystal data		
Chemical formula	C_18_H_21_NO_3_	C_16_H_17_NO_3_
*M*_r_	299.36	271.30
Crystal system, space group	Monoclinic, *P*2_1_/*c*	Orthorhombic, *P* *b* *c* *a*
Temperature (K)	90	90
*a*, *b*, *c* (Å)	11.4982 (7), 5.0086 (3), 26.7240 (16)	11.8747 (10), 7.2485 (6), 30.392 (2)
α, β, γ (°)	90, 97.683 (2), 90	90, 90, 90
*V* (Å^3^)	1525.22 (16)	2616.0 (4)
*Z*	4	8
Radiation type	Mo *K*α	Mo *K*α
μ (mm^−1^)	0.09	0.10
Crystal size (mm)	0.58 × 0.07 × 0.07	0.28 × 0.06 × 0.06

Data collection		
Diffractometer	Bruker D8 goniometer	Bruker D8 goniometer
Absorption correction	Multi-scan (*SADABS*; Bruker, 2018 ▸)	Multi-scan (*SADABS*; Bruker, 2018 ▸)
*T*_min_, *T*_max_	0.580, 0.747	0.666, 0.746
No. of measured, independent and observed [*I* > 2σ(*I*)] reflections	27741, 4862, 4204	41504, 3506, 2193
*R*_int_	0.066	0.128
(sin θ/λ)_max_ (Å^−1^)	0.725	0.685

Refinement		
*R*[*F* ^2^ > 2σ(*F* ^2^)], *wR*(*F* ^2^), *S*	0.049, 0.121, 1.07	0.050, 0.143, 1.05
No. of reflections	4862	3506
No. of parameters	202	182
H-atom treatment	H atoms treated by a mixture of independent and constrained refinement	H-atom parameters constrained
Δρ_max_, Δρ_min_ (e Å^−3^)	0.42, −0.26	0.28, −0.26

Computer programs: *APEX3* and *SAINT* (Bruker, 2018 ▸), *SHELXT2014/5* (Sheldrick, 2015*a* ▸), *SHELXL2018/3* (Sheldrick, 2015*b* ▸) and *ShelXle* (Hübschle *et al.*, 2011 ▸).

## supplementary crystallographic information

### 5-(2H-1,3-Benzodioxol-5-yl)-N-cyclohexylpenta-2,4-dienamide (I) Crystal data

**Table:**

C_18_H_21_NO_3_	*F*(000) = 640
*M**_r_* = 299.36	*D*_x_ = 1.304 Mg m^−^^3^
Monoclinic, *P*2_1_/*c*	Mo *K*α radiation, λ = 0.71073 Å
*a* = 11.4982 (7) Å	Cell parameters from 9948 reflections
*b* = 5.0086 (3) Å	θ = 2.5–33.5°
*c* = 26.7240 (16) Å	µ = 0.09 mm^−^^1^
β = 97.683 (2)°	*T* = 90 K
*V* = 1525.22 (16) Å^3^	Needle, light-yellow
*Z* = 4	0.58 × 0.07 × 0.07 mm

### 5-(2H-1,3-Benzodioxol-5-yl)-N-cyclohexylpenta-2,4-dienamide (I) Data collection

**Table:**

Bruker D8 goniometer diffractometer	4862 independent reflections
Radiation source: microfocus X-ray tube	4204 reflections with *I* > 2σ(*I*)
Multilayered conforacal mirror monochromator	*R*_int_ = 0.066
Detector resolution: 7.391 pixels mm^-1^	θ_max_ = 31.0°, θ_min_ = 2.2°
ω scans	*h* = −16→16
Absorption correction: multi-scan (SADABS; Bruker, 2018)	*k* = −7→7
*T*_min_ = 0.580, *T*_max_ = 0.747	*l* = −38→38
27741 measured reflections	

### 5-(2H-1,3-Benzodioxol-5-yl)-N-cyclohexylpenta-2,4-dienamide (I) Refinement

**Table:**

Refinement on *F*^2^	Primary atom site location: dual
Least-squares matrix: full	Hydrogen site location: mixed
*R*[*F*^2^ > 2σ(*F*^2^)] = 0.049	H atoms treated by a mixture of independent and constrained refinement
*wR*(*F*^2^) = 0.121	*w* = 1/[σ^2^(*F*_o_^2^) + (0.0455*P*)^2^ + 0.7464*P*] where *P* = (*F*_o_^2^ + 2*F*_c_^2^)/3
*S* = 1.07	(Δ/σ)_max_ = 0.001
4862 reflections	Δρ_max_ = 0.42 e Å^−^^3^
202 parameters	Δρ_min_ = −0.26 e Å^−^^3^
0 restraints	

### 5-(2H-1,3-Benzodioxol-5-yl)-N-cyclohexylpenta-2,4-dienamide (I) Special details

**Table:**

Geometry. All esds (except the esd in the dihedral angle between two l.s. planes) are estimated using the full covariance matrix. The cell esds are taken into account individually in the estimation of esds in distances, angles and torsion angles; correlations between esds in cell parameters are only used when they are defined by crystal symmetry. An approximate (isotropic) treatment of cell esds is used for estimating esds involving l.s. planes.

### 5-(2H-1,3-Benzodioxol-5-yl)-N-cyclohexylpenta-2,4-dienamide (I) Fractional atomic coordinates and isotropic or equivalent isotropic displacement parameters (Å^2^)

**Table:**

	*x*	*y*	*z*	*U*_iso_*/*U*_eq_	
O1	0.96864 (7)	0.23821 (16)	0.65434 (3)	0.01577 (17)	
O2	0.26072 (7)	0.95788 (18)	0.47532 (3)	0.01982 (18)	
O3	0.09239 (7)	0.7586 (2)	0.49784 (4)	0.0246 (2)	
N1	1.02983 (8)	0.66732 (19)	0.66764 (4)	0.01464 (18)	
H1	1.0072 (13)	0.834 (3)	0.6656 (6)	0.018*	
C1	1.35763 (10)	0.7868 (2)	0.69502 (4)	0.0179 (2)	
H1A	1.387593	0.633931	0.677037	0.021*	
H1AB	1.404140	0.946479	0.688562	0.021*	
C2	1.37316 (10)	0.7289 (2)	0.75164 (4)	0.0177 (2)	
H2A	1.456652	0.689410	0.763536	0.021*	
H2AB	1.350298	0.887869	0.770054	0.021*	
C3	1.29754 (11)	0.4914 (2)	0.76259 (4)	0.0184 (2)	
H3A	1.306633	0.458942	0.799445	0.022*	
H3AB	1.324548	0.329830	0.746168	0.022*	
C4	1.16802 (10)	0.5412 (2)	0.74321 (4)	0.0174 (2)	
H4A	1.121475	0.381401	0.749567	0.021*	
H4AB	1.139004	0.693282	0.761708	0.021*	
C5	1.15165 (9)	0.6024 (2)	0.68667 (4)	0.01204 (19)	
H5	1.174757	0.440743	0.668389	0.014*	
C6	1.22876 (10)	0.8346 (2)	0.67461 (4)	0.0156 (2)	
H6A	1.201602	1.000303	0.689604	0.019*	
H6AB	1.220984	0.859459	0.637563	0.019*	
C7	0.13448 (10)	0.9561 (2)	0.46564 (4)	0.0178 (2)	
H00F	0.103160	1.134167	0.472821	0.021*	
H00G	0.108313	0.912274	0.429779	0.021*	
C8	0.29070 (10)	0.7663 (2)	0.51139 (4)	0.0146 (2)	
C9	0.19016 (9)	0.6451 (2)	0.52444 (4)	0.0157 (2)	
C10	0.19552 (10)	0.4395 (2)	0.55871 (4)	0.0172 (2)	
H10	0.126694	0.356487	0.567425	0.021*	
C11	0.30804 (10)	0.3586 (2)	0.58014 (4)	0.0154 (2)	
H11	0.315407	0.213891	0.603318	0.018*	
C12	0.40980 (9)	0.4841 (2)	0.56850 (4)	0.0135 (2)	
C13	0.40115 (9)	0.6936 (2)	0.53284 (4)	0.0142 (2)	
H13	0.469038	0.780458	0.524074	0.017*	
C14	0.52399 (9)	0.3949 (2)	0.59388 (4)	0.0151 (2)	
H14	0.528504	0.219194	0.607404	0.018*	
C15	0.62312 (9)	0.5413 (2)	0.59963 (4)	0.0153 (2)	
H15	0.618873	0.720765	0.588024	0.018*	
C16	0.73553 (9)	0.4407 (2)	0.62246 (4)	0.0144 (2)	
H16	0.741526	0.256496	0.630909	0.017*	
C17	0.83199 (9)	0.5927 (2)	0.63240 (4)	0.0147 (2)	
H17	0.825437	0.779183	0.626212	0.018*	
C18	0.94810 (9)	0.4819 (2)	0.65259 (4)	0.01252 (19)	

### 5-(2H-1,3-Benzodioxol-5-yl)-N-cyclohexylpenta-2,4-dienamide (I) Atomic displacement parameters (Å^2^)

**Table:**

	*U*^11^	*U*^22^	*U*^33^	*U*^12^	*U*^13^	*U*^23^
O1	0.0151 (4)	0.0087 (3)	0.0230 (4)	0.0010 (3)	0.0008 (3)	0.0012 (3)
O2	0.0130 (4)	0.0226 (4)	0.0237 (4)	0.0022 (3)	0.0019 (3)	0.0074 (3)
O3	0.0116 (4)	0.0333 (5)	0.0284 (5)	0.0015 (4)	0.0014 (3)	0.0109 (4)
N1	0.0124 (4)	0.0082 (4)	0.0228 (5)	0.0014 (3)	0.0006 (3)	0.0001 (3)
C1	0.0146 (5)	0.0186 (5)	0.0199 (5)	−0.0044 (4)	0.0007 (4)	0.0004 (4)
C2	0.0181 (5)	0.0134 (5)	0.0201 (5)	0.0006 (4)	−0.0034 (4)	−0.0003 (4)
C3	0.0222 (5)	0.0137 (5)	0.0181 (5)	0.0006 (4)	−0.0016 (4)	0.0024 (4)
C4	0.0191 (5)	0.0176 (5)	0.0160 (5)	−0.0002 (4)	0.0037 (4)	0.0016 (4)
C5	0.0112 (4)	0.0089 (4)	0.0158 (5)	0.0010 (3)	0.0012 (3)	0.0004 (3)
C6	0.0160 (5)	0.0119 (5)	0.0181 (5)	−0.0033 (4)	−0.0007 (4)	0.0031 (4)
C7	0.0140 (5)	0.0196 (5)	0.0195 (5)	0.0029 (4)	0.0010 (4)	0.0003 (4)
C8	0.0149 (5)	0.0148 (5)	0.0144 (5)	0.0007 (4)	0.0035 (4)	−0.0001 (4)
C9	0.0106 (4)	0.0198 (5)	0.0167 (5)	0.0007 (4)	0.0020 (4)	−0.0022 (4)
C10	0.0128 (5)	0.0208 (5)	0.0183 (5)	−0.0038 (4)	0.0035 (4)	−0.0010 (4)
C11	0.0148 (5)	0.0160 (5)	0.0156 (5)	−0.0028 (4)	0.0031 (4)	0.0003 (4)
C12	0.0121 (4)	0.0134 (5)	0.0153 (5)	−0.0005 (4)	0.0030 (3)	−0.0015 (4)
C13	0.0117 (4)	0.0145 (5)	0.0169 (5)	−0.0008 (4)	0.0037 (4)	0.0006 (4)
C14	0.0137 (5)	0.0148 (5)	0.0169 (5)	0.0016 (4)	0.0030 (4)	0.0016 (4)
C15	0.0138 (5)	0.0138 (5)	0.0183 (5)	0.0025 (4)	0.0027 (4)	0.0006 (4)
C16	0.0144 (5)	0.0129 (5)	0.0162 (5)	0.0023 (4)	0.0029 (4)	0.0011 (4)
C17	0.0138 (5)	0.0110 (4)	0.0194 (5)	0.0030 (4)	0.0023 (4)	0.0020 (4)
C18	0.0127 (4)	0.0108 (4)	0.0143 (5)	0.0006 (4)	0.0027 (3)	0.0010 (3)

### 5-(2H-1,3-Benzodioxol-5-yl)-N-cyclohexylpenta-2,4-dienamide (I) Geometric parameters (Å, º)

**Table:**

O1—C18	1.2429 (13)	C6—H6A	0.9900
O2—C8	1.3710 (14)	C6—H6AB	0.9900
O2—C7	1.4400 (14)	C7—H00F	0.9900
O3—C9	1.3705 (14)	C7—H00G	0.9900
O3—C7	1.4372 (15)	C8—C13	1.3710 (15)
N1—C18	1.3440 (14)	C8—C9	1.3909 (15)
N1—C5	1.4614 (13)	C9—C10	1.3741 (16)
N1—H1	0.874 (16)	C10—C11	1.4028 (15)
C1—C2	1.5275 (16)	C10—H10	0.9500
C1—C6	1.5279 (16)	C11—C12	1.3991 (15)
C1—H1A	0.9900	C11—H11	0.9500
C1—H1AB	0.9900	C12—C13	1.4120 (15)
C2—C3	1.5246 (17)	C12—C14	1.4648 (15)
C2—H2A	0.9900	C13—H13	0.9500
C2—H2AB	0.9900	C14—C15	1.3468 (15)
C3—C4	1.5305 (16)	C14—H14	0.9500
C3—H3A	0.9900	C15—C16	1.4442 (15)
C3—H3AB	0.9900	C15—H15	0.9500
C4—C5	1.5284 (15)	C16—C17	1.3423 (15)
C4—H4A	0.9900	C16—H16	0.9500
C4—H4AB	0.9900	C17—C18	1.4793 (15)
C5—C6	1.5228 (15)	C17—H17	0.9500
C5—H5	1.0000		
			
C8—O2—C7	105.94 (9)	H6A—C6—H6AB	107.9
C9—O3—C7	106.11 (9)	O3—C7—O2	107.98 (9)
C18—N1—C5	123.36 (9)	O3—C7—H00F	110.1
C18—N1—H1	116.8 (10)	O2—C7—H00F	110.1
C5—N1—H1	119.9 (10)	O3—C7—H00G	110.1
C2—C1—C6	111.27 (9)	O2—C7—H00G	110.1
C2—C1—H1A	109.4	H00F—C7—H00G	108.4
C6—C1—H1A	109.4	C13—C8—O2	127.72 (10)
C2—C1—H1AB	109.4	C13—C8—C9	122.25 (10)
C6—C1—H1AB	109.4	O2—C8—C9	110.03 (10)
H1A—C1—H1AB	108.0	O3—C9—C10	128.10 (10)
C3—C2—C1	110.13 (9)	O3—C9—C8	109.91 (10)
C3—C2—H2A	109.6	C10—C9—C8	121.98 (10)
C1—C2—H2A	109.6	C9—C10—C11	116.43 (10)
C3—C2—H2AB	109.6	C9—C10—H10	121.8
C1—C2—H2AB	109.6	C11—C10—H10	121.8
H2A—C2—H2AB	108.1	C12—C11—C10	122.19 (11)
C2—C3—C4	111.23 (9)	C12—C11—H11	118.9
C2—C3—H3A	109.4	C10—C11—H11	118.9
C4—C3—H3A	109.4	C11—C12—C13	119.88 (10)
C2—C3—H3AB	109.4	C11—C12—C14	118.98 (10)
C4—C3—H3AB	109.4	C13—C12—C14	121.13 (10)
H3A—C3—H3AB	108.0	C8—C13—C12	117.22 (10)
C5—C4—C3	110.66 (9)	C8—C13—H13	121.4
C5—C4—H4A	109.5	C12—C13—H13	121.4
C3—C4—H4A	109.5	C15—C14—C12	125.40 (10)
C5—C4—H4AB	109.5	C15—C14—H14	117.3
C3—C4—H4AB	109.5	C12—C14—H14	117.3
H4A—C4—H4AB	108.1	C14—C15—C16	123.65 (11)
N1—C5—C6	108.34 (9)	C14—C15—H15	118.2
N1—C5—C4	111.98 (9)	C16—C15—H15	118.2
C6—C5—C4	111.37 (9)	C17—C16—C15	123.70 (10)
N1—C5—H5	108.3	C17—C16—H16	118.1
C6—C5—H5	108.3	C15—C16—H16	118.1
C4—C5—H5	108.3	C16—C17—C18	122.76 (10)
C5—C6—C1	111.66 (9)	C16—C17—H17	118.6
C5—C6—H6A	109.3	C18—C17—H17	118.6
C1—C6—H6A	109.3	O1—C18—N1	123.06 (10)
C5—C6—H6AB	109.3	O1—C18—C17	122.66 (10)
C1—C6—H6AB	109.3	N1—C18—C17	114.26 (9)

### 5-(2H-1,3-Benzodioxol-5-yl)-N-cyclohexylpenta-2,4-dienamide (I) Hydrogen-bond geometry (Å, º)

**Table:**

*D*—H···*A*	*D*—H	H···*A*	*D*···*A*	*D*—H···*A*
N1—H1···O1^i^	0.874 (16)	2.086 (16)	2.9547 (12)	172.8 (14)

Symmetry code: (i) *x*, *y*+1, *z*.

### 5-(2H-1,3-Benzodioxol-5-yl)-1-(pyrrolidin-1-yl)penta-2,4-dien-1-one (II) Crystal data

**Table:**

C_16_H_17_NO_3_	*D*_x_ = 1.378 Mg m^−^^3^
*M**_r_* = 271.30	Mo *K*α radiation, λ = 0.71073 Å
Orthorhombic, *P**b**c**a*	Cell parameters from 3874 reflections
*a* = 11.8747 (10) Å	θ = 2.7–25.8°
*b* = 7.2485 (6) Å	µ = 0.10 mm^−^^1^
*c* = 30.392 (2) Å	*T* = 90 K
*V* = 2616.0 (4) Å^3^	Needle, colorless
*Z* = 8	0.28 × 0.06 × 0.06 mm
*F*(000) = 1152	

### 5-(2H-1,3-Benzodioxol-5-yl)-1-(pyrrolidin-1-yl)penta-2,4-dien-1-one (II) Data collection

**Table:**

Bruker D8 goniometer diffractometer	3506 independent reflections
Radiation source: microfocus X-ray tube	2193 reflections with *I* > 2σ(*I*)
Multilayered conforacal mirror monochromator	*R*_int_ = 0.128
Detector resolution: 7.391 pixels mm^-1^	θ_max_ = 29.1°, θ_min_ = 2.7°
ω scans	*h* = −16→16
Absorption correction: multi-scan (SADABS; Bruker, 2018)	*k* = −9→9
*T*_min_ = 0.666, *T*_max_ = 0.746	*l* = −41→41
41504 measured reflections	

### 5-(2H-1,3-Benzodioxol-5-yl)-1-(pyrrolidin-1-yl)penta-2,4-dien-1-one (II) Refinement

**Table:**

Refinement on *F*^2^	Hydrogen site location: inferred from neighbouring sites
Least-squares matrix: full	H-atom parameters constrained
*R*[*F*^2^ > 2σ(*F*^2^)] = 0.050	*w* = 1/[σ^2^(*F*_o_^2^) + (0.0492*P*)^2^ + 1.9223*P*] where *P* = (*F*_o_^2^ + 2*F*_c_^2^)/3
*wR*(*F*^2^) = 0.143	(Δ/σ)_max_ = 0.001
*S* = 1.05	Δρ_max_ = 0.28 e Å^−^^3^
3506 reflections	Δρ_min_ = −0.26 e Å^−^^3^
182 parameters	Extinction correction: SHELXL-2018/3 (Sheldrick 2015b), Fc^*^=kFc[1+0.001xFc^2^λ^3^/sin(2θ)]^-1/4^
0 restraints	Extinction coefficient: 0.0038 (6)

### 5-(2H-1,3-Benzodioxol-5-yl)-1-(pyrrolidin-1-yl)penta-2,4-dien-1-one (II) Special details

**Table:**

Geometry. All esds (except the esd in the dihedral angle between two l.s. planes) are estimated using the full covariance matrix. The cell esds are taken into account individually in the estimation of esds in distances, angles and torsion angles; correlations between esds in cell parameters are only used when they are defined by crystal symmetry. An approximate (isotropic) treatment of cell esds is used for estimating esds involving l.s. planes.

### 5-(2H-1,3-Benzodioxol-5-yl)-1-(pyrrolidin-1-yl)penta-2,4-dien-1-one (II) Fractional atomic coordinates and isotropic or equivalent isotropic displacement parameters (Å^2^)

**Table:**

	*x*	*y*	*z*	*U*_iso_*/*U*_eq_	
O1	0.90586 (12)	0.3421 (2)	0.67241 (5)	0.0219 (3)	
O2	0.77314 (12)	0.9757 (2)	0.35830 (4)	0.0239 (4)	
O3	0.62806 (12)	0.8589 (2)	0.40118 (5)	0.0238 (3)	
N1	0.72232 (13)	0.3304 (2)	0.69173 (5)	0.0178 (4)	
C1	0.80525 (16)	0.3650 (3)	0.66249 (6)	0.0179 (4)	
C2	0.77084 (17)	0.4355 (3)	0.61881 (6)	0.0200 (4)	
H2	0.693195	0.435608	0.611273	0.024*	
C3	0.84580 (17)	0.4994 (3)	0.58954 (6)	0.0195 (4)	
H3	0.923497	0.493700	0.596862	0.023*	
C4	0.81458 (18)	0.5762 (3)	0.54755 (6)	0.0202 (4)	
H4	0.737486	0.571321	0.539278	0.024*	
C5	0.88758 (17)	0.6543 (3)	0.51932 (6)	0.0204 (4)	
H5	0.964674	0.654787	0.527665	0.024*	
C6	0.85964 (17)	0.7386 (3)	0.47701 (6)	0.0189 (4)	
C7	0.94619 (18)	0.8085 (3)	0.45072 (7)	0.0233 (5)	
H7	1.021461	0.801352	0.461170	0.028*	
C8	0.92613 (18)	0.8889 (3)	0.40946 (7)	0.0238 (5)	
H8	0.985763	0.934891	0.391755	0.029*	
C9	0.81622 (17)	0.8975 (3)	0.39603 (6)	0.0197 (4)	
C10	0.65583 (18)	0.9250 (3)	0.35806 (7)	0.0243 (5)	
H10A	0.608744	1.033210	0.350559	0.029*	
H10B	0.642106	0.827357	0.335911	0.029*	
C11	0.72958 (17)	0.8288 (3)	0.42176 (6)	0.0192 (4)	
C12	0.74749 (17)	0.7485 (3)	0.46174 (6)	0.0194 (4)	
H12	0.686800	0.701056	0.478668	0.023*	
C13	0.74989 (17)	0.2759 (3)	0.73696 (6)	0.0191 (4)	
H13A	0.803365	0.364049	0.750477	0.023*	
H13B	0.783182	0.150743	0.737737	0.023*	
C14	0.63666 (17)	0.2799 (3)	0.76059 (7)	0.0214 (4)	
H14A	0.633146	0.183869	0.783722	0.026*	
H14B	0.622901	0.401963	0.774158	0.026*	
C15	0.55179 (17)	0.2411 (3)	0.72417 (7)	0.0229 (5)	
H15A	0.476464	0.289608	0.731974	0.028*	
H15B	0.545712	0.107030	0.718398	0.028*	
C16	0.59994 (16)	0.3421 (3)	0.68437 (7)	0.0202 (4)	
H16A	0.578131	0.280454	0.656583	0.024*	
H16B	0.574336	0.472052	0.683550	0.024*	

### 5-(2H-1,3-Benzodioxol-5-yl)-1-(pyrrolidin-1-yl)penta-2,4-dien-1-one (II) Atomic displacement parameters (Å^2^)

**Table:**

	*U*^11^	*U*^22^	*U*^33^	*U*^12^	*U*^13^	*U*^23^
O1	0.0160 (7)	0.0250 (8)	0.0247 (7)	0.0012 (6)	−0.0006 (6)	0.0005 (6)
O2	0.0236 (8)	0.0286 (8)	0.0195 (7)	−0.0012 (6)	−0.0007 (6)	0.0050 (6)
O3	0.0196 (8)	0.0307 (8)	0.0209 (7)	0.0019 (6)	−0.0009 (6)	0.0048 (6)
N1	0.0134 (8)	0.0218 (9)	0.0181 (8)	−0.0001 (7)	−0.0008 (6)	0.0024 (7)
C1	0.0177 (10)	0.0158 (9)	0.0204 (10)	−0.0005 (8)	−0.0011 (8)	−0.0021 (8)
C2	0.0186 (10)	0.0206 (10)	0.0208 (10)	0.0005 (8)	−0.0025 (8)	−0.0014 (8)
C3	0.0199 (10)	0.0194 (10)	0.0191 (10)	0.0002 (8)	−0.0009 (8)	−0.0017 (8)
C4	0.0201 (10)	0.0201 (10)	0.0203 (10)	0.0012 (8)	−0.0022 (8)	−0.0017 (8)
C5	0.0181 (10)	0.0214 (10)	0.0216 (10)	0.0014 (8)	−0.0003 (8)	−0.0002 (8)
C6	0.0187 (10)	0.0197 (10)	0.0184 (10)	0.0013 (8)	0.0002 (8)	−0.0022 (8)
C7	0.0185 (10)	0.0283 (11)	0.0230 (10)	−0.0001 (9)	0.0006 (8)	0.0003 (9)
C8	0.0214 (11)	0.0265 (11)	0.0236 (10)	−0.0015 (9)	0.0048 (8)	0.0028 (9)
C9	0.0232 (11)	0.0204 (10)	0.0156 (9)	−0.0011 (8)	0.0008 (8)	0.0006 (8)
C10	0.0218 (11)	0.0308 (12)	0.0204 (10)	0.0001 (9)	−0.0006 (8)	0.0026 (9)
C11	0.0180 (10)	0.0197 (10)	0.0200 (10)	0.0009 (8)	−0.0011 (8)	−0.0004 (8)
C12	0.0182 (10)	0.0202 (10)	0.0197 (9)	−0.0001 (8)	0.0025 (8)	−0.0001 (8)
C13	0.0198 (10)	0.0196 (10)	0.0180 (9)	0.0017 (8)	−0.0007 (8)	0.0008 (8)
C14	0.0206 (10)	0.0218 (10)	0.0218 (10)	0.0007 (9)	0.0021 (8)	0.0008 (8)
C15	0.0171 (10)	0.0266 (11)	0.0251 (10)	−0.0011 (9)	0.0018 (8)	−0.0016 (9)
C16	0.0144 (9)	0.0241 (11)	0.0221 (10)	0.0011 (8)	−0.0007 (8)	0.0000 (8)

### 5-(2H-1,3-Benzodioxol-5-yl)-1-(pyrrolidin-1-yl)penta-2,4-dien-1-one (II) Geometric parameters (Å, º)

**Table:**

O1—C1	1.243 (2)	C7—H7	0.9500
O2—C9	1.377 (2)	C8—C9	1.369 (3)
O2—C10	1.441 (2)	C8—H8	0.9500
O3—C11	1.376 (2)	C9—C11	1.385 (3)
O3—C10	1.434 (2)	C10—H10A	0.9900
N1—C1	1.350 (2)	C10—H10B	0.9900
N1—C13	1.467 (2)	C11—C12	1.364 (3)
N1—C16	1.473 (2)	C12—H12	0.9500
C1—C2	1.480 (3)	C13—C14	1.525 (3)
C2—C3	1.341 (3)	C13—H13A	0.9900
C2—H2	0.9500	C13—H13B	0.9900
C3—C4	1.441 (3)	C14—C15	1.523 (3)
C3—H3	0.9500	C14—H14A	0.9900
C4—C5	1.345 (3)	C14—H14B	0.9900
C4—H4	0.9500	C15—C16	1.525 (3)
C5—C6	1.462 (3)	C15—H15A	0.9900
C5—H5	0.9500	C15—H15B	0.9900
C6—C7	1.397 (3)	C16—H16A	0.9900
C6—C12	1.412 (3)	C16—H16B	0.9900
C7—C8	1.403 (3)		
			
C9—O2—C10	104.98 (15)	O2—C10—H10A	110.2
C11—O3—C10	105.50 (15)	O3—C10—H10B	110.2
C1—N1—C13	120.26 (16)	O2—C10—H10B	110.2
C1—N1—C16	127.52 (16)	H10A—C10—H10B	108.5
C13—N1—C16	112.21 (15)	C12—C11—O3	127.55 (19)
O1—C1—N1	121.10 (18)	C12—C11—C9	122.74 (19)
O1—C1—C2	121.93 (18)	O3—C11—C9	109.69 (17)
N1—C1—C2	116.95 (17)	C11—C12—C6	117.50 (19)
C3—C2—C1	122.08 (19)	C11—C12—H12	121.3
C3—C2—H2	119.0	C6—C12—H12	121.3
C1—C2—H2	119.0	N1—C13—C14	103.86 (16)
C2—C3—C4	123.4 (2)	N1—C13—H13A	111.0
C2—C3—H3	118.3	C14—C13—H13A	111.0
C4—C3—H3	118.3	N1—C13—H13B	111.0
C5—C4—C3	124.2 (2)	C14—C13—H13B	111.0
C5—C4—H4	117.9	H13A—C13—H13B	109.0
C3—C4—H4	117.9	C15—C14—C13	103.75 (16)
C4—C5—C6	126.23 (19)	C15—C14—H14A	111.0
C4—C5—H5	116.9	C13—C14—H14A	111.0
C6—C5—H5	116.9	C15—C14—H14B	111.0
C7—C6—C12	119.16 (18)	C13—C14—H14B	111.0
C7—C6—C5	119.19 (18)	H14A—C14—H14B	109.0
C12—C6—C5	121.64 (18)	C14—C15—C16	103.87 (16)
C6—C7—C8	122.5 (2)	C14—C15—H15A	111.0
C6—C7—H7	118.8	C16—C15—H15A	111.0
C8—C7—H7	118.8	C14—C15—H15B	111.0
C9—C8—C7	116.59 (19)	C16—C15—H15B	111.0
C9—C8—H8	121.7	H15A—C15—H15B	109.0
C7—C8—H8	121.7	N1—C16—C15	102.81 (16)
C8—C9—O2	128.41 (18)	N1—C16—H16A	111.2
C8—C9—C11	121.54 (19)	C15—C16—H16A	111.2
O2—C9—C11	110.01 (18)	N1—C16—H16B	111.2
O3—C10—O2	107.62 (16)	C15—C16—H16B	111.2
O3—C10—H10A	110.2	H16A—C16—H16B	109.1

## References

[bb1] Bruker (2018). *APEX3*, *SAINT* and *SADABS*. Bruker AXS Inc., Madison, Wisconsin, USA.

[bb2] Ezawa, T., Inoue, Y., Murata, I., Takao, K., Sugita, Y. & Kanamoto, I. (2018). *AAPS PharmSciTech*, **19**, 923–933. 10.1208/s12249-017-0908-9 29071656

[bb3] Ezawa, T., Inoue, Y., Murata, I., Takao, K., Sugita, Y. & Kanamoto, I. (2019). *Int. J. Med. Chem.* **2019**, 1–14. 10.1155/2019/7530480 PMC6388355 30886749

[bb4] Ezawa, T., Inoue, Y., Tunvichien, S., Suzuki, R. & Kanamoto, I. (2016). *Int. J. Med. Chem.* **2016**, 1–9. 10.1155/2016/8723139 PMC4779834 26998357

[bb5] Hübschle, C. B., Sheldrick, G. M. & Dittrich, B. (2011). *J. Appl. Cryst.* **44**, 1281–1284. 10.1107/S0021889811043202 PMC3246833 22477785

[bb6] Pfund, L. Y., Chamberlin, B. L. & Matzger, A. J. (2015). *Cryst. Growth Des.* **15**, 2047–2051. 10.1021/acs.cgd.5b00278 PMC5436616 28529462

[bb7] Sheldrick, G. M. (2015*a*). *Acta Cryst.* A**71**, 3–8.

[bb8] Sheldrick, G. M. (2015*b*). *Acta Cryst.* C**71**, 3–8.

[bb9] Szejtli, J. (1998). *Chem. Rev.* **98**, 1743–1754. 10.1021/cr970022c 11848947

[bb10] Takao, K., Miyashiro, T. & Sugita, Y. (2015). *Chem. Pharm. Bull.* **63**, 326–333. 10.1248/cpb.c14-00874 25948326

